# The Influence of the Microstructure of Ceramic-Elastomer Composites on Their Energy Absorption Capability

**DOI:** 10.3390/ma14216618

**Published:** 2021-11-03

**Authors:** Paulina Kozera, Anna Boczkowska, Rafał Kozera, Marcin Małek, Włodzimierz Idczak

**Affiliations:** 1 Faculty of Materials Science and Engineering, Warsaw University of Technology, ul. Woloska 141, 02-507 Warsaw, Poland; anna.boczkowska@pw.edu.pl (A.B.); rafal.kozera@pw.edu.pl (R.K.); 2 Faculty of Civil Engineering and Geodesy, Military University of Technology, ul. Gen. Sylwestra Kaliskiego 2, 00-908 Warsaw, Poland; marcin.malek@wat.edu.pl (M.M.); wlodzimierz.idczak@wat.edu.pl (W.I.)

**Keywords:** interpenetrating phase composites, ceramic preform, static and dynamic tests, specific surface fraction of the interphase boundaries, energy absorption capability, strain rate, stress at plateau zone

## Abstract

The paper presents the experimental results of static and dynamic compressive tests conducted on ceramic-elastomer composites. The alumina ceramic preforms were fabricated by the four-step method: ceramic mixture preparation, consolidation under pressure, presintering, and sintering under pressure, respectively. To obtain ceramic preforms with a similar volume fraction of open pores, but with different pore sizes, alumina powder with different particle size and a ceramic binder were used, as well as pore-forming agents that were evenly distributed throughout the volume of the molding mass. The composites were obtained using vacuum pressure infiltration of porous alumina ceramic by urea-urethane elastomer in liquid form. As a result, the obtained composites were characterized by two phases that interpenetrated three-dimensionally and topologically throughout the microstructure. The microstructure of the ceramic preforms was revealed by X-ray tomography, which indicated that the alumina preforms had similar porosity of approximately 40% vol. but different pore diameter in the range of 6 to 34 µm. After composite fabrication, image analysis was carried out. Due to the microstructure of the ceramic preforms, the composites differed in the specific surface fraction of the interphase boundaries (S_v_). The highest value of the S_v_ parameter was achieved for composite fabricated by infiltration method of using ceramic preform with the smallest pore size. Static and dynamic tests were carried out using different strain rate: 1.4·10^−3^, 7·10^−2^, 1.4·10^−1^, and 3·10^3^ s^−1^. Compressive strength, stress at plateau zone, and absorbed energy were determined. It was found that the ceramic-elastomer composites’ ability to absorb energy depended on the specific surface fraction of the interphase boundaries and achieved a value between 15.3 MJ/m^3^ in static test and 51.1 MJ/m^3^ for dynamic strain rate.

## 1. Introduction

As a result of the constantly growing demand for lightweight materials with improved physicochemical, mechanical, and thermal properties, the development of new composites can be observed. Composite materials called interpenetrating phase composites (IPCs) or co-continuous composites may have properties that are unattainable by other materials. In the IPCs, matrix and reinforcement are interconnected in all three spatial dimensions [1,2].

Many different strategies have been proposed in the literature to process co-continuous composites including powder metallurgy [1], squeeze casting [2,3], and pressure or non-pressure liquid infiltration using open-cell preforms [4,5,6] as well as reactive infiltration [7,8]. The possibility of using different fabrication methods determines the wide range of materials that make up interconnected phases in IPCs and various mechanical and macrostructural characteristics. Jhaver and Tippur [4,6] infiltrated an open-cell aluminum preform by uncured epoxy-based syntactic foam. Cylindrical IPC foam specimens were tested in uniaxial compression and failure responses were examined. The IPC foam samples in general and the silane coated ones in particular showed significant improvement in elastic modulus, yield stress, energy absorption, and plateau stress values when compared to the corresponding syntactic foam of the same volume fraction of micro balloons. Liu and Li [9,10] modelled the mechanical properties of the interpenetrating phase composites of aluminum foam/polyurethane. It was observed that the compression strength of AF/PU composites increased with the increasing volume fraction of aluminum, while the failure mode of AF/PU composites is irregular due to the introduction of PU.

In [11] work, the mechanical properties of novel types of 3D-printed interpenetrating phase composites (IPCs) with periodic architectures were investigated. The IPCs consisted of a hard solid phase that reinforced a softer phase, where both phases are made of polymers. The results of the uniaxial compressive test showed that while the hard phase endures a larger fraction of the load, the softer phase confines cracks and prevents catastrophic failure.

Prielipp et al. [12] and Chou et al. [13] focused on the mechanical properties of Al/Al_2_O_3_ composites with interpenetrating networks. It was found that the increase in the fracture strength of the composites depended on crack through the matrix and the initiation of the crack initiation at metal-filled pores.

Several researcher results have confirmed that co-continuous ceramic-polymer composites are a group of advanced materials characterized by high fracture toughness, isotropic properties, and the ability to achieve large deformations [14,15,16,17]. As a result of the combination of stiff ceramic and flexible polymer, it is possible to obtain a composite with the capacity to absorb mechanical energy [18,19].

Based on compression test results, the character of stress–strain curves for ceramic-polymer composites and aluminum foams is similar [20,21]. A typical strain-stress curve of aluminum foams contains a non-linear elastic zone (with small plastic deformations), a quasi-linear plateau zone, and a non-linear material compaction zone. Aluminum foams are used as shock absorbers because of their high relative energy absorption. Similarly, elastic deformation has occurred in the initial zone in IPCs [22,23,24]. The ceramic preform is responsible for the transmission of stress and, as a result, micro cracks appeared in the porous ceramic structure. Macroscopic cracks do not appear at this stage. When maximum strength is achieved, the stress decreases. This is related to exceeding the critical density of micro cracks in the ceramic preform microstructure [25,26]. Not only are micro-cracks formed in ceramic grains, but above all, cracks appear in the entire porous structure of the ceramic matrix. In this area, called the plateau zone, the ceramic skeleton is further fragmented, which suggests a further decrease in stress. In this zone, however, the elastomer is responsible for the transmission of stress. The re-increase of stress is closely related to the compaction of the material, resulting from the action of compressive loads [9,14,24]. The properties of composites strongly depend on their microstructure, especially the pores of the shape and size of the ceramic preform pores, type and strength of the phase connection at the interface, and defects occurring during composite fabrication, such as voids, air bubbles, and cracks in the ceramic structure. In addition, it is important to achieve a continuity of both phases relative to each other and a high degree of filling the pores of the preform by the liquid phase.

The mechanical properties of composites with an interpenetrating phases structure depend on the durability of the connection of both components. The strength of the phase separation surface, i.e., the interphase boundary, depends on the type of phase connection, the appropriate wettability of one material by the other, and the surface roughness of the filled component. The structure of the interface is also a very important factor [25,26,27].

In the case of SiO_2_-elastomer IPCs [14], the share of interface boundaries is very important. The increase in the fracture toughness of the composite occurs through braking or deflecting a crack of ceramics in the vicinity elastomer. In the compression test, the ceramics break first; then the stresses are transferred by the elastomer, which buckles. Cracks can also “extinguish” on the elastomer due to stress relaxation. The stress acting on the elastomer causes a change in the conformation of the macroparticles, which enables the deformation to be the sum of the elastic deformation and the highly elastic and plastic deformations to increase over time. As the highly elastic and plastic strains increase, the elastic strains decrease over time, which results in a reduction in the acting stresses. Therefore, science and industry are interested in the participation and role of the interface boundaries in IPCs [28].

This paper presents the influence of ceramic-elastomer composites microstructure on their energy absorption capability. The IPCs were fabricated using vacuum-pressure infiltration of porous alumina ceramic by the urea-urethane elastomer. Because the ceramic preforms are characterized by 40 vol.% porosity and different pore sizes, four types of IPCs were obtained. Based on image analysis results, the specific surface fraction of the interphase boundaries for all composites was determined. In this paper, the results of the compressive tests have been presented in this paper depending on the specific surface fraction of the interphase boundaries.

## 2. Materials and Methods

### 2.1. Materials

The sintering method of ceramic particles is less commonly used. Usually, for the fabrication of ceramic preforms, chemical or mechanical foaming of the ceramic mass, as well as techniques of the plastic cellular structure mapping, is used. These methods make possible the production of ceramic preforms with a porosity of not less than 70%. In addition, the obtained structures are very fragile. Attempts to obtain a smaller contribution of pores by these methods are associated with the introduction of additional processes and quite often lead to the formation of closed pores, which prevents the introduction of the liquid phase into them [29,30,31].

The four types of ceramic preforms were fabricated by the four-step method in the Institute of Ceramics and Building Materials consisting of ceramic mixture preparation, uniaxial compaction, pre-sintering, and hot isostatic pressing (HIP). To allow ceramic forms to be characterized by the same value of porosity but different pore size, four different size alumina powders supplied by the P.P.U.H.KOS company were used. The alumina powders were mixed with 10÷15 wt % of Granulox NM9922 (Nabaltec AG, Schwandorf, Germany) high-temperature ceramic binder and 7 wt % of dextrin solution as pore structure forming agent. As a consequence, four different mixtures were obtained and their composition was shown in Table 1.

**Figure 1:**
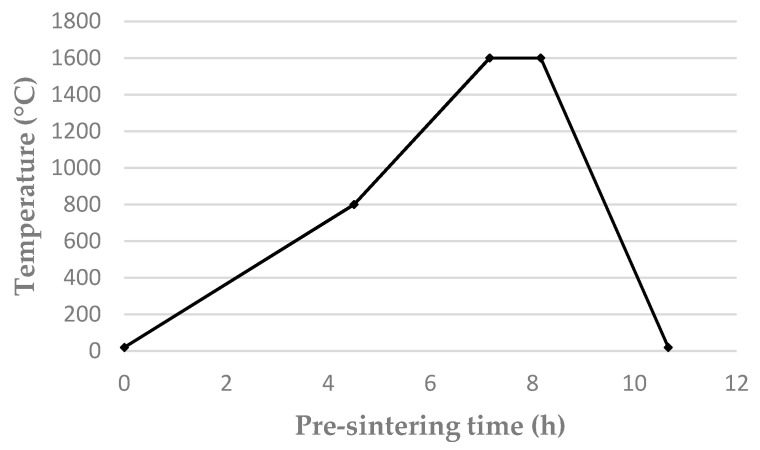
Temperature curve of the ceramic preforms pre-sintering process.

After the preparation of four types of ceramic mixtures, they were inserted into steel forms and molded by uniaxial pressing at a working pressure of MPa. In the next step, the presintering of semi-finished preforms was performed using an electric chamber furnace according to the set process parameters presented in Figure 1.

Finally, in order to enhance mechanical properties and increase the density of ceramic preforms, hot isostatic pressing was conducted under 200 MPa pressure and in 1600 °C temperature for one hour.

The ceramic-elastomer composites were made by infiltration of the ceramic preforms with a reactive mixture of substrates in liquid form. Urea urethane elastomers (PU2.5) were synthesized by a one-shot method from 4,4′-methylenebis (phenylisocyanate) (MDI), poly(ethylene adipate) (PEA), and dicyandiamide (DCDA). The molar ratio of MDI/(PEA + DCDA) substrates was equal to 2.5 (which means hard to soft segments ratio of 1.50).

### 2.2. Methods

The microstructure of ceramic preforms was revealed using the Sky-Scan 1174 X-ray tomography (SkyScan, Aartselaar, Belgium). Before scanning, samples in the shape of a cuboid with dimensions 10 × 10× 15 mm did not require any special preparation. Scanning was performed using an X-ray tube with the following parameters: 100 kV voltage, 100 kA, no filter material, 0.5° rotation step in an angle interval of 180°. The obtained cross-sections of the ceramic preforms were studied using CTAn software (Billerica, MA, USA) and as a result the volume fraction of both phases and the pore size were determined.

The application of the micrometer image analysis program made possible the investigation of the specific surface fraction of the interphase boundaries in the composites. Stereological analysis was carried out using SEM images of composites performed by Hitachi TM3000 scanning electron microscopy (SEM) (HITACHI High-Technologies Corporation, Tokyo, Japan).

Static compressive tests were carried out using the MTS Q/Test 10 test machine (MTS Testing Systems, Toronto, ON, Canada) according to the ISO 20504:2019 standard [32]. To exhibit the strain rate sensitivity of IPCs, which was mainly attributed to the rate effect of foams or preforms, different strain rates were applied. The tests were carried out with three velocities: 1, 50, and 100 mm/min, which gives an initial strain rate $\dot{\varepsilon }$ equal to 1.4·10^−3^, 7·10^−2^, and 1.4·10^−1^ s^−1^, respectively.

**Figure 2:**
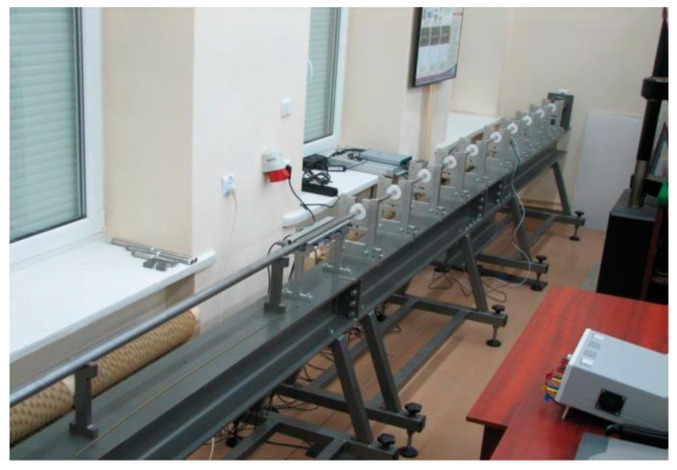
Station for measuring materials at high strain rates.

The dynamic behavior of the composites was investigated using the split Hopkinson pressure bar technique (SHPB) in conjunction with high-speed photography. A dynamic compression test was conducted using the elastic bar system (modified Hopkinson split pressure bar system) at the Military University of Technology presented in Figure 2. The signals of the bars were amplified using the LTT500 Tasler amplifier (LTT Labortechnik Tasler GmbH, Würzburg, Germany). The Hopkinson split pressure bar was characterized not only by its strain excitation system, but mainly by its measurement system, which attempted to eliminate elastic wave interference. The impact speed was a variable value and was about 3·10^3^ s^−1^.

**Figure 3:**
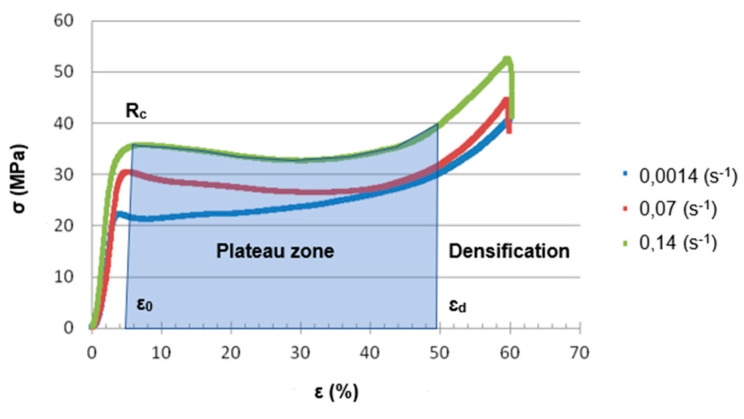
The stress–strain curves for Al_2_O_3_/PU2.5 composites with an explanation of how the compressive strength (R_c_), stress at plateau zone (σ_pl_), and absorbed energy (EA) were calculated.

Based on the obtained stress–strain curves, the compressive strength, stress in the plateau zone, and absorbed energy were determined. Three deformation stages can be observed on each stress–strain curve of the ceramic elastomer composites presented in Figure 3. First is the elastic stage, between 0% and approx. 5% of failure strain. Second plateau stage, which starts from approximately 5% of failure strain and ends with the densification strain. For this stage, slight stress increase together with a rapidly increased strain can be observed [33]. The last stage is the densification stage. In this study, the plateau stress $\sigma_{pl}$ was expressed as Equation (1):

(1) $$\sigma_{pl}=\frac{1}{\varepsilon_{d}-\varepsilon_{0}}\int_{\varepsilon_{0}}^{\varepsilon_{d}}\sigma d\varepsilon$$

where 0 was the failure strain of the ceramic preform and $\varepsilon_{d}$ was the densification strain which corresponds to the turning point from the plateau stage to the densification stage on the stress–strain curve as well as the extreme value on the energy absorption efficiency [33].

Energy absorption *EA* (*ε*) was expressed as Equation (2):

(2) $$EA\;\left(\varepsilon \right)=\int_{0}^{\varepsilon }\sigma d\varepsilon$$

## 3. Results and Discussion

### 3.1. X-ray Tomography of Ceramic Preforms

**Figure 4:**
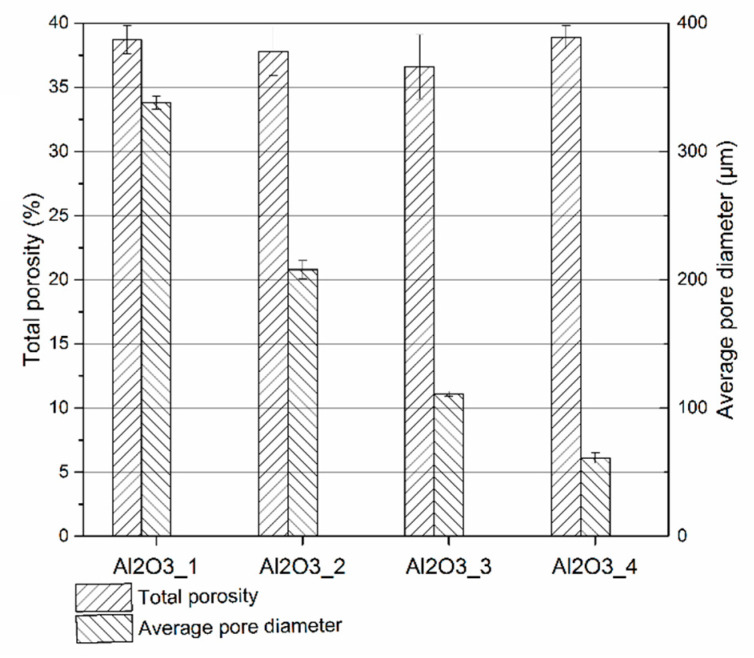
Total porosity and average pore diameter for Al_2_O_3__1, Al_2_O_3__2, Al_2_O_3__3, and Al_2_O_3__4 ceramic preforms.

**Figure 5:**
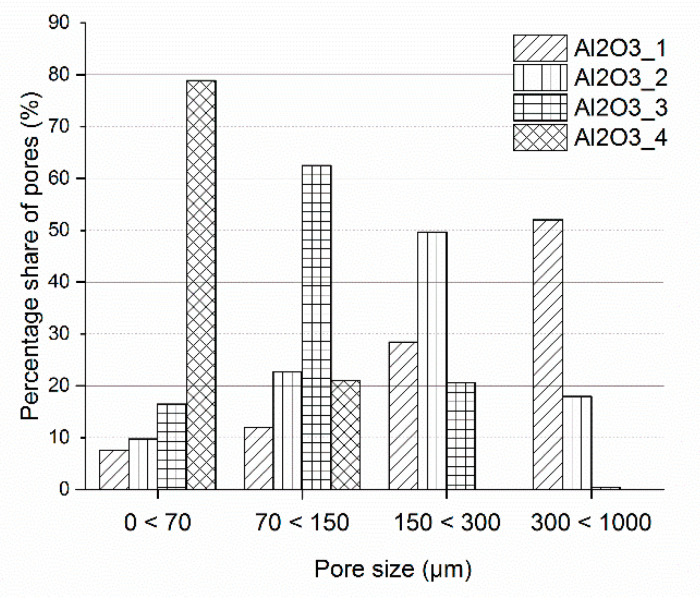
Pore size distribution of Al_2_O_3__1, Al_2_O_3__2, Al_2_O_3__3, and Al_2_O_3__4 ceramic preforms.

**Figure 6:**
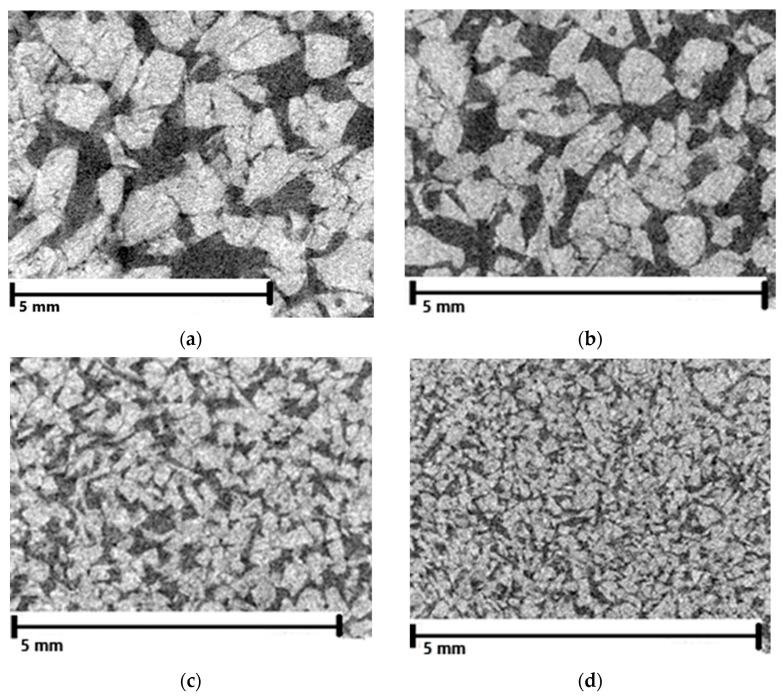
X-ray tomography images for preforms with similar porosity and different pore sizes designated as (**a**) Al_2_O_3__1, (**b**) Al_2_O_3__2, (**c**) Al_2_O_3__3, and (**d**) Al_2_O_3__4, respectively.

The ceramic preforms were tested using computed x-ray tomography. Results of the average pore’s diameter and total porosity of the preforms are presented in Figure 4, while the pore’s size distribution is presented in Figure 5. In addition, two-dimensional photos of scanned areas of ceramic preforms are shown in Figure 6. It was found that proper selected ceramic mixture composition, alumina particles size as well as the fabrication method of ceramic preforms made it possible to obtain four types of preforms with similar porosity of approx. 40 vol.%. These performs were different in the average pore size and in the fraction of pores of a certain size. The average pore diameter of the ceramic Al_2_O_3__1 ceramic preform was 34 µm whereas for Al_2_O_3__4 it was only 6 µm. The change in the pore size of ceramic preforms with the same porosity was achieved by using different sizes of ceramic particles for preform fabrication.

It was reported that these preforms also differ in their specific pore’s surface. This parameter determines the total surface of the pores located in the volume unit of the porous preform. The higher the specific surface area of the ceramic pores, the greater the contribution of an interface in the ceramic-elastomer composites.

Moreover, as can be observed in Figure 6, the ceramic particles possess irregular shapes and sharp edges. They touch each other to form a ceramic skeleton. The pores of each preform differ in shape. Generally, pores can be divided into open and closed, and due to their shape, into cylindrical open at both ends and blind in the shape of an inkwell, funnel, or incision [33]. In the case of the preforms produced in this work, we observed all these shapes of pores.

### 3.2. Stereological Analysis of Composites

**Figure 7:**
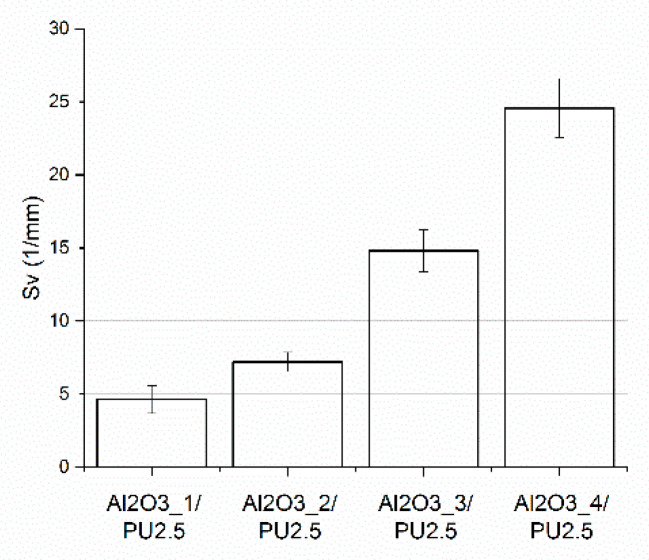
The specific surface fraction S_v_ of the interphase boundaries of Al_2_O_3__1/PU2.5, Al_2_O_3__2/PU2.5, Al_2_O_3__3/PU2.5, and Al_2_O_3__4/PU2.5 composites.

**Figure 8:**
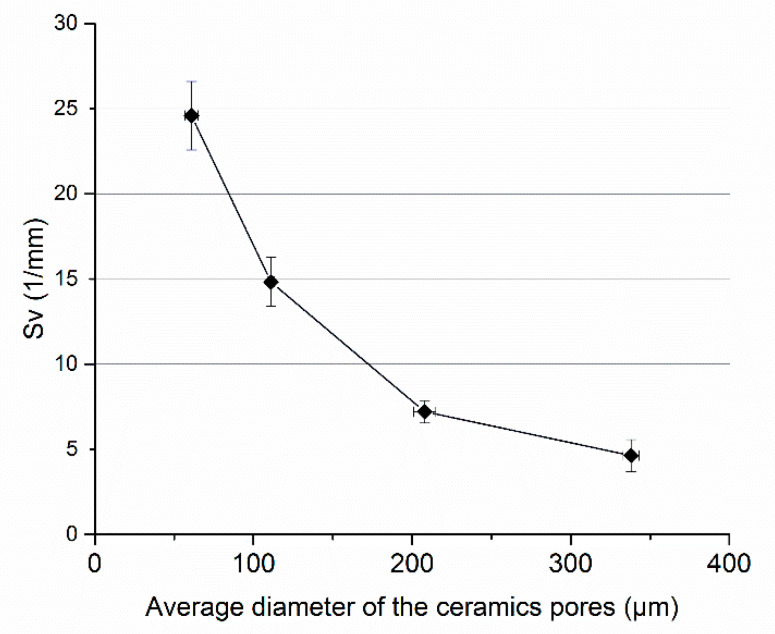
The specific surface fraction S_v_ of the interphase boundaries of composites depending on the pore size of the ceramic preform.

Stereological analysis of composites allowed to determine the specific surface fraction of the interphase boundaries. The results are presented in Figure 7 and Figure 8. It was indicated that the specific surface fraction of the interphase boundaries increases when the pore size of the preforms from which the composites were made decreases. The composites fabricated using ceramic preforms with the smallest diameter of pores were characterized by the highest S_v_ parameter. For example, for the Al_2_O_3__4/PU2.5 composites, obtained via the infiltration of ceramic preforms with an average pore size below 70 µm, the S_v_ value was determined and amounted to 24.59 1/mm. The authors of the work [15,34] received similar results.

### 3.3. Static and Dynamic Strength Tests

The results calculated from the stress–strain curves for the composites achieved from static and dynamic compressive tests are shown in Table 2.

The compressive strength, the stress at plateau, and energy absorbed by the composite depend on the strain rate. It was observed that, the higher the compressive speed, the higher the strength parameters of the composites. For all types of composites, the compressive strength increased five-fold as a result of using a dynamic strain rate. The same tendency can be observed in the case of stress at plateau as well as energy absorption. The tested composite materials showed sensitivity to strain rate, especially during the dynamic tests, which proved that the materials can work as shock absorbers. This is also confirmed by the fact that the EA energy values obtained for ceramic-elastomer composites with phase percolation are higher than the energy values of energy absorbed by aluminum foams [35]. However, the obtained curves are not linear. The results presented in [9,36] confirm similar relationships for ceramic-polymer composites.

The strain rate effects on compressive strength and plateau stress of IPCs have been widely reported [24,33]. In the case of composites based on aluminum foams, four reasons for the strain rate effects on mechanical properties were reported: the flow effect of the pore fluid, the strain rate sensitivity of the matrix materials, enhancement of shock waves, and the microinertia effects of the microstructure. In this study, polyurethane elastomers were characterized by deformation highly flexible and strain-rate sensitivity.

**Figure 9:**
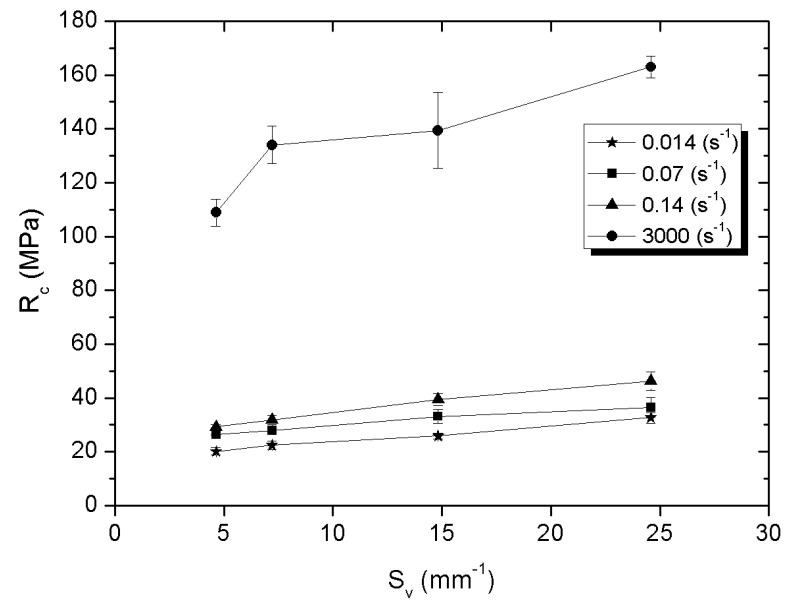
Compressive strength of ceramic-elastomer composites depending on S_v_ parameter for 1.4·10^−3^, 7·10^−2^, 1.4·10^−1^, and 1·10^3^ s^−1^ strain velocity.

**Figure 10:**
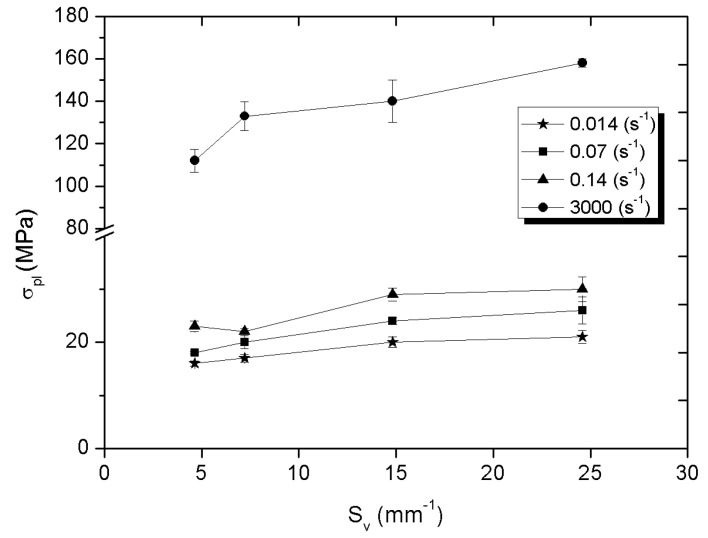
Plateau stress of ceramic-elastomer composites depending on S_v_ parameter for 1.4·10^−3^, 7·10^−2^, 1.4·10^−1^, and 1·10^3^ s^−1^ strain rate.

**Figure 11:**
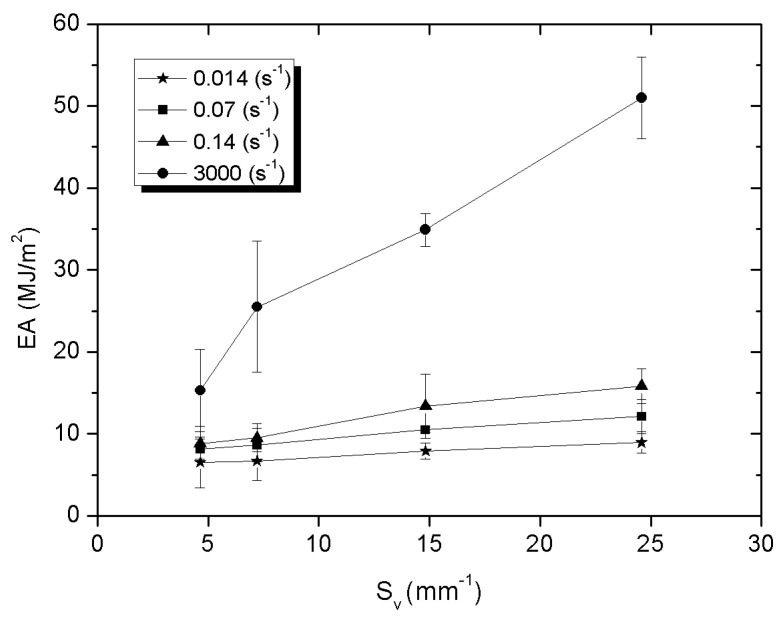
Absorbed energy of ceramic-elastomer composites depending on S_v_ parameter for 1.4·10^−3^, 7·10^−2^, 1.4·10^−1^, and 1·10^3^ s^−1^ strain rate.

Figure 9, Figure 10 and Figure 11 present the influence of the specific surface fraction of the interphase boundaries on compressive strength, stress at the plateau, and the value of absorbed energy determined for all composites and for all applied strain rate.

It was also found that the mechanical properties of Al_2_O_3_-elastomer composites with phase percolation strongly depend on the specific surface fraction of the interphase boundaries. Compressive strength, stress at plateau and absorbed energy increase as the number of interphase boundaries grows. In the case of static strain rates, this character of the curves is similar to a linear function, while for a higher value of ε the nature of the curves is more logarithmic. The Al_2_O_3__4/PU2.5 composite with the highest specific surface fraction of the interphase boundaries is characterized by the highest compressive strength, stress at plateau, and the ability to absorb energy, regardless of the rate of strain. Such dependence may result from the fact that the high S_v_ parameter value causes the deformed elastomer to be more strongly limited by the higher amount of ceramic walls. As a result of compressive loads, high stresses arise in the elastomer structure. This mechanism can be compared to the method of strengthening the material with grain boundaries [35].

Differences in compressive strength, stress at plateau, and absorbed energy for the same strain rate mainly depend on the properties of the ceramic matrix and the effect of the surface expansion. According to [16] study, in ceramics with the smallest particles due to the capillary effects necks have the relatively greatest width and their amount per unit of volume quantity is higher. Hence composites fabricated using ceramics sintered from the smallest powder fraction grains showed the highest strength.

## 4. Conclusions

The four types of alumina/elastomer interpenetrating phase composites (IPCs) were fabricated by filling the polyurethane elastomer in liquid form into the ceramic porous. The ceramic preforms used in the fabrication of composites differed in the size of pores with the same porosity. As a result, the composites differed by the specific surface fraction of the interphase boundaries. The static and dynamic compression tests were performed to study the mechanical response in terms of strength, stress at a plateau, and energy absorption properties. The results were summarized as follows:

1. The applied method of producing ceramic samples allowed to obtain preforms with 40% of porosity and different pore sizes.

2. The specific surface fraction of the interphase boundaries has an impact on the ceramic-elastomer composite’s ability to absorb energy, as well as on compressive strength and stress at the plateau zone at both static and dynamic compressions.

3. The composites, fabricated using the preforms obtained by sintering the smallest sizes of alumina particles, were characterized by the highest S_v_ value.

4. The ceramic-elastomer IPCs showed strain rate sensitivity. For all types of composites, the compressive strength, stress at the plateau, and ability to absorb energy increased as a result of using a dynamic strain rate.

5. The ceramic-elastomer composites in which two phases are interpenetrating three-dimensionally and topologically throughout the microstructure can be applied as shock absorbers.

## Figures and Tables

**Table 1:** Composition of the ceramic mixture used for the fabrication of forms.

Ceramic Mixture/Preform Name	Alumina Particles Size (µm)	Weight Fraction of Granulox NM9922 Binder (%)	Weight Fraction of Dextrin Solution (%)
Al_2_O_3__1	1400–1200	10 ÷ 15	7
Al_2_O_3__2	1200–1000		
Al_2_O_3__3	600–300		
Al_2_O_3__4	300–100		

**Table 2:** Mechanical properties of composites determined during static and dynamic compressive tests.

Material/S_v_	$\mathrm{Strain}\;\mathrm{Rate}\;\dot{\varepsilon }$ (s^−1^)	Compressive Strength R_c_ (MPa)	Stress at Plateau *σ_pl_* (MPa)	Absorbed Energy EA (MJ/m^3^)
Al_2_O_3__1/PU2.5 Composite 4.64 (1/mm)	1.4·10^−3^	20.07 ± 1.37	40.13 ± 6.9	6.53 ± 0.12
	7·10^−2^	26.37 ± 0.23	19.03 ± 4.0	8.18 ± 0.24
	1.4·10^−1^	29.30 ± 0.81	45.34 ± 6.6	8.82 ± 1.09
	3·10^3^	108.8 ± 5.3	>110.5	15.3 ± 0.9
Al_2_O_3__2/PU2.5 Composite 7.21 (1/mm)	1.4·10^−3^	22.40 ± 1.70	17.4 ± 1.10	6.71 ± 0.43
	7·10^−2^	27.87 ± 1.19	20.1 ± 1.30	8.63 ± 1.03
	1.4·10^−1^	31.80 ± 1.70	22.8 ± 2.30	9.52 ± 0.70
	3·103	134.00 ± 7.5	133.5 ± 4.6	25.5 ± 0.5
Al_2_O_3__3/PU2.5 Composite 14.83 (1/mm)	1.4·10^−3^	25.80 ± 1.00	20.1 ± 0.90	7.9 ± 0.97
	7·10^−2^	33.15 ± 2.52	24.7 ± 0.87	10.49 ± 0.33
	1.4·10^−1^	39.43 ± 2.11	29.4 ± 1.50	13.4 ± 0.92
	3·10^3^	139.40 ± 14.9	140.9 ± 2.90	34.91 ± 0.9
Al_2_O_3__4/PU2.5 Composite 24.59 (1/mm)	1.4·10^−3^	32.65 ± 1.97	21.0 ± 0.70	8.94 ± 1.31
	7·10^−2^	36.48 ± 3.68	26.1 ± 2.10	12.11 ± 1.11
	1.4·10^−1^	46.23 ± 3.35	30.3 ± 4.90	15.83 ± 1.12
	3·10^3^	162.9 ± 4.1	158.5 ± 8.1	51.1 ± 2.09

## Data Availability

The data presented in this study are available upon request from the corresponding author.
